# Hepatocellular Carcinomas with Granulomatous Inflammation In Tumor Stroma: Clinicopathologic Characteristics

**DOI:** 10.5146/tjpath.2021.01562

**Published:** 2022-05-19

**Authors:** Burcu Saka, Ferhat Ozden, Ayse Armutlu, Gokhan Ertugrul

**Affiliations:** Department of Pathology, Istanbul Medipol University, School of Medicine, Istanbul, Turkey; Koc University, School of Medicine, Istanbul, Turkey; Department of General Surgery, Istanbul Medipol University, School of Medicine, Istanbul, Turkey

**Keywords:** Hepatocellular carcinoma, Inflammation, Granuloma

## Abstract

*
Objective:* To determine the frequency of granulomatous inflammation within hepatocellular carcinoma (HCC) and its clinicopathologic associations.

*
Material and Method:* Fifty-eight HCCs (51 explants, 3 lobectomies, and 4 segmentectomies) were reviewed.

*
Results:* Five (8.6%) cases (F/M=1/4, mean age: 63.6) were identified with granulomas.1/5 had history of neoadjuvant therapy. 4/5 patients presented with early stage (pT1/2). All were well-differentiated (Grade1-2/4). The mean number of tumor foci was 3.6, with a median size of 2.2 cm. All of them had advanced fibrosis. No difference was identified from cases without granulomas (n=53) in terms of prognosis and aforementioned parameters (p> 0.05). Granulomas were mainly concentrated in peripheral parts of the tumors. One case with nodule-in-nodule formation had granulomas lined along the border of the inner nodule. In 2 cases, granulomas were identified in steatohepatitic areas, while another had clear cell change. Only 1 had necrotizing granulomas, none with acid resistant bacilli. Two cases revealed concomitant granulomas in the adjacent liver parenchyma in addition to the tumor stroma. Except for one with a history of tuberculosis, none of the cases had a granulomatous disease.

*
Conclusion:* This is the largest case series of HCCs with granulomas by far. Our data revealed neither clinicopathologic and prognostic difference nor definite etiology related to granulomas. Yet, association with steatotic and clear tumor cells suggests the role of cytoplasmic content, while distribution of granulomas points to host immune response.

## INTRODUCTION

Granulomatous inﬂammation is a unique type of chronic inﬂammatory response (1). Although it does not indicate a definite etiology, its detection limits the differential diagnosis list leading to effective treatment. Granulomas may be associated with infectious [e.g. tuberculosis (Tbc)] or noninfectious diseases (e.g. sarcoidosis, Crohn’s disease) and local irritants (e.g. necrotic material) (1). Tumor-related granuloma stays as a rare etiology in this differential diagnosis list.

Malignancy related granulomatous inflammation was observed as early as 1911 and throughout the time, it has been defined mainly in 3 locations; in tumor draining lymph nodes (LNs), distant organs, or within tumor stroma (2–5). ‘Sarcoid-like reaction’ is a commonly used term for all these 3 forms, but is mainly preferred to define a systemic inflammatory response resembling sarcoidosis in both clinical and pathologic aspects.

Hodgkin lymphoma and disgerminoma/seminoma (in about 15% of cases) constitute well-known examples of malignancies characterized by granulomas in tumor stroma (6). They are followed by some rare types of malignancies; mainly carcinomas but also a few sarcomas (7,8). Hepatocellular carcinoma (HCC) is one of these rare carcinomas represented by only 4 case reports in the last 40 years (9–12), when patients with comorbidities such as sarcoidosis and Tbc are excluded (13–15). There is virtually no systematic analysis regarding the frequency and clinicopathologic characteristics of HCC cases harbouring granulomatous inflammation.

This study aimed to determine the frequency of granulo-matous inflammation in HCCs (‘granulomatous cases’), as well as to define the clinicopathologic features of these cases. We also aimed to determine the etiology and prognostic impact of this reaction by comparing them with HCCs without granulomas (‘non granulomatous cases’).

## MATERIALS and METHODS

### Cases

Consecutive 58 hepatic resections (51 explants, 3 lobecto-mies and 4 segmentectomies) diagnosed as HCC, approximately in a period of five years were included.

### Definitions

Nonspesific histiocytic reactions such as histiyocytes around tumor necrosis, microsphere-related foreign body reactions, or isolated histiyocytic giant cells dispersed in tumor stroma were excluded. Collection of activated macrophages (epithelioid cells) were recorded as ‘granuloma’ (1). Accompanying cells (lymphocytes, eosinophils, Langhans giant cells) and necrosis were also recorded. Small, uniform, discrete, naked granulomas without necrosis were accepted as ‘sarcoid-like’ granuloma (1).

### Histopathological and Histochemical Analysis

All Hematoxylin-eosin slides were reviewed by a single observer. Whenever needed for conflicting parameters, two pathologists decided together.

All the relevant parameters required to determine pT (AJCC 8th ed.) were noted in additon to tumor size, histologic grade (based on Edmondson and Steiner grading system), histologic subtype, and tumor necrosis of five largest foci (16,17). Granulomas and their characteristics; distribution throughout the tumor (in the center or at the periphery), presence of necrosis, accompanying inflammatory cells (lymphocytes and eosinophils), and Langhans type giant cells were reviewed. Intratumoral inflammation (apart from granulomatous inflammation) was screened at 10x and arbitrarily scored as none, minimal (barely perceptible), and moderate/dense (easily perceptible).

Slides of background liver (57 of 58) and regional LNs of dissected cases (16 of 58) were also examined regarding fibrotic stage and granulomatous inflammation.

Ziehl-Neelsen staining was performed in each tumor block with stromal granulomas.

### Evaluation of Clinical Parameters

Information on the patients’ gender, age, etiology of chronic liver disease, history of neoadjuvant therapy and follow-up information were obtained through pathology databases, patients’ charts and national database of death certificates. The patients who died within the first 30 days of the postoperative period (9 patients in non granulomatous group) were excluded from the survival analysis.

### Statistical Analysis

The analysis was performed using The jamovi project (2021, Version 1.6, Computer Software, Retrieved from https://www.jamovi.org.) and R Core Team (2020, Version 4.0, Computer software, Retrieved from https://cran.r-project.org.). R packages retrieved from MRAN snapshot 2020-08-24.

Clinicopathological variables were compared according to the presence of granulomas. Since the granulomatous cases’ group was low in number, continuous variables were compared with the Mann-Whitney U Test, and proportions of categorical variables were compared with Fisher’s Exact Test. Phi-coefficient and Cramer-V tests were used to assess the strength of association.

Clinical outcomes were recorded and analyzed by Kaplan-Meier curves, and the differences in clinicopathological features and overall survival between groups compared by log-rank analysis.

### Ethical Aspects

The study was conducted in full accordance with local GCP guideline and current legislations, while the permission was obtained from the institutional ethics committee (Date: 7.17.2019, Approval number: 583) for the use of patient data for publication purposes.

## RESULTS

### Incidence

Among 58 cases of HCC, intratumoral granulomatous inflammation was identified in 5 (8.6%).

### Clinicopathologic Features of the Study Cohort

The patients were four males and one female [F/M=0.25, vs 0.2 in cases without (w/o) granulomas]. The mean age was 63.6 years (vs. 57.2 in HCC w/o granulomas) (Table 1).

Of the 5 cases with granuloma, 2 had Hepatitis B, 1 had Hepatitis C, 1 had non-alcoholic steatohepatitis, and 1 had multiple factors (Hepatitis B and alcohol) leading to cirrhosis. The non-granulomatous group had similar etiological distribution, as viral hepatitis was the main cause of chronic liver disease. In contrast to granulomatous ones, approximately one-tenth (7.6 %) of the non-granulomatous cases were devoid of advanced fibrosis. The proportion of patients with neoadjuvant therapy was roughly similar between the two groups (20% in granulomatous and 28% in non-granulomatous cases). All the patients in study cohort were organ confined (Table 1).

**Table 1:** Clinicopathological features of 5 cases with granulomas and 53 cases w/o granulomas comparatively.

		**HCC with granulomas**	**HCC without granulomas**	**p value**
Age	Mean (SD)	63.6 (4.0)	57.2 (11.6)	0.091*
Gender [n, (%)]	Female	1 (10)	9 (90)	1.0**
	Male	4 (8)	44 (92)	
Etiology of chronic liver disease^1^ [n, (%)]	Hepatitis B	2 (11)	16 (89)	0.974**
	Hepatitis C	1 (13)	7 (88)	
	Alcohol	-	7(100)	
	Nonalcoholic steatohepatitis	1 (14)	6 (86)	
	Cryptogenic	-	3 (100)	
	Multiple factors	1 (17)	5 (83)	
Advanced fibrosis (Ishak Score 5-6/6)^2^ [n, (%)]	Present	4 (8)	48 (92)	0.379**
	Absent	1 (20)	4 (80)	
History of neoadjuvant therapy [n, (%)]	Present	1 (7)	14 (93)	1.00**
	Absent	4 (9)	39 (91)	
Numbers of tumor foci	Mean (SD)	3.6 (2.1)	2.6 (1.9)	0.251*
	Median (min-max)	4 (1-6)	2 (1-6)	
Tumor size^3^	Mean (cm) (SD)	3.9 (3.8)	4.8 (4.4)	0.729*
	Median (min-max)	2.2 (1-4)	3.2 (0.5-19)	
Histologic grade^3^ [n, (%)]	1	1 (17)	5 (83)	0.443**
	2	4 (11)	34 (89)	
	3	-	12 (100)	
	4	-	2 (100)	
Macrovascular invasion [n, (%)]	Present	-	5 (100)	1.00**
	Absent	5 (9)	47 (91)	
Microvascular invasion [n, (%)]	Present	2 (11)	17 (89)	1.00**
	Absent	3 (8)	36 (92)	
Intratumoral inflammation [n, (%)]	Moderate/Dense	1 (7)	13 (93)	0.584**
	Minimal	4 (13)	26 (87)	
	Absent	-	14 (100)	
Necrosis in tumor [n, (%)]	Present	2 (7)	28 (93)	0.665**
	Absent	3 (11)	25 (89)	
Outcome [n, (%)]	Alive	3 (6)	44 (94)	0.237**
	Dead	2 (18)	9 (82)	

*Mann-Whitney U Test, **Fisher Exact Test **HCC:** Hepatocellular carcinoma, **CC:** Cholangiocarcinoma, **^1^:** Budd-Chiari syndrome (1 case), Glycogen storage disease (1 case) and 7 N/A cases in the nongranulomatous group are not listed here, **^2^:** not accessible for 1 case in the nongranulomatous group, **^3^:** features of greatest focus in multifocal cases

Mean numbers of tumor foci were 3.6 and 2.6 in granulomatous and non-granulomatous cases, respectively. Mean and median tumor sizes of granulomatous cases were smaller (3.9 cm vs. 4.75 and 2.2 cm vs. 3.2 cm in non-granulomatous cases), although not reached statistical significant difference (Table 1).

Totally 131 tumor nodules were investigated in 58 cases, while 17 of them were noted in 5 granulomatous cases. Four of 5 cases were multifocal, with 1 of 4 (Case 1), 2 of 5 (Case 3), 3 of 5 (Case 4) and 2 of 2 (Case 5) tumor foci with granulomas. Collectively, granulomas were detected in 9 tumor foci (Table 2).

**Table 2:** Clinicopathologic details of 5 HCC cases with intratumoral granulomas.

**Case**	**Age**	**Sex**	**Etiology of chronic liver disease**	Tumor size* (cm)	**No. of tumor foci**	**No. of tumor foci w granulomas**	**pT**	**Follow-up (Overall survival)**	**Medical** **history**	**Granuloma(s) was/were identified**
1	58	M	HBV+ Alcohol	1.8	4	1	2	Exitus (9 mos)	Tbc, DM, CVD	-in a single focus with SH subtype
2	67	M	NASH	1.3	1	1	1a	Alive (44 mos)	CAD	
3	67	M	HBV	10.4	5	2	3	Exitus (33 mos)	DM	-in 2 of 3 foci with clear cell change - in the background liver, adjacent to tumor
4	61	F	HCV	2.2	5	3	2	Alive (21 mos)***	HT	- in the background liver, adjacent to one focus - in confluent pattern in one focus - densely in area with SH features in one focus
5	65	M	HBV	4	2	2	2	Alive (11.5 mos)	**-**	- on the pseudocapsule of inner nodule in one focus **- **with necrosis surrounded by palisading histiocytes in another focus

**CAD:** Coronary artery disease, **CVD:** Cerebrovascular disease, **DM:** Diabetes mellitus, **F:** Female, **HBV:** Hepatitis B, **HCC:** Hepatocellular carcinoma, **HCV:** Hepatitis C, **M:** Male, **NASH:** Nonalcoholic steatohepatitis, **No:** Number, **SH:** Steatohepatitic, *: The size of largest focus in multifocal cases, ***: Metastasis of humerus detected at 18th month

All of granulomatous cases were lower histologic grade (1 was grade 1/4 and remaining were grade 2/4) compared to non-granulomatous ones, in which 17 were (29 %) grade 3-4/4 (Table 1).

Regarding histologic subtypes of 17 foci in 5 granulomatous cases, two foci (Case 1 and dominant focus of Case 3) were the steatohepatitic (SH) subtype (more than 5% of tumor represents SH features) while another (one focus of Case 4) was characterised with focal (less than 5%) SH features. SH characteristics (16 cases with> 5% and 1 case with <%5) were identified in totally 17 of 114 tumor foci investigated in 53 HCC w/o granulomas. Of note, 3 foci of 114 foci were clear cell subtype (> 50% of tumor with clear cells), while 8 of them had clear cell features (<50% of tumor with clear cells).

The macrovascular invasion was not identified in granulomatous cases, in contrast to one-tenth of non-granulomatous ones (5 of 53, 9.4%). The microvascular invasion was detected 40% and 32% of cases, respectively in granulomatous and non-granulomatous groups.

Except for one case, all granulomatous cases presented with early stage (pT1/2) in comparison with 74% of HCC cases w/o granulomas.

No statistically significant difference was found between the groups regarding these documented features (Table 1).

No drug history was identified except for anti-hypertensive and anti-diabetic medications. Only 1 of 5 patient was treated for Tbc 7 years before (Case 1), with sequel changes at the apex of the lung. Of note, this was a nonnecrotizing case (Table 2).

### Histopathologic Details of Granulomatous Inflammation

**Figure 1:**
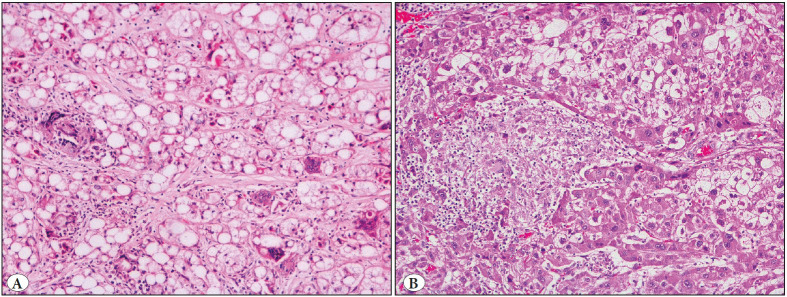
**A)** Stetohepatitic hepatocellular carcinoma with dispersed histiocytic giant cells forms a granuloma in the left upper quadrant of the image (Case 1), H&E, X20 **B)** Granuloma in relation to tumor cells with clear cytoplasm (Two foci of Case 3), H&E, x20.

Granulomas were localized mainly in circumferential regions of tumor stroma (n=6), within ~2 mm (approximately 10x objective diameter) from the tumor/nontumor interface, even very rarely in touch with tumor pseudocapsule (Case 2). In 2 foci with steatohepatitis-like features, granulomas were concentrated specifically in these areas, instead of tumor periphery (Case 1 and 4, Figure 1A) while intermingled with clear cells in two foci (Case 3, Figure 1B). One focus with nodule-in-nodule formation had multiple granulomas located on the fibrotic pseudocapsule surrounding the inner nodule (Case 5). This focus also had granulomas at the peripheral part of the outer nodule.

**Figure 2:**
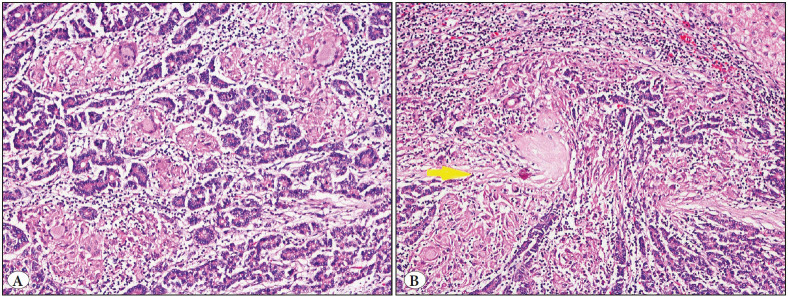
One focus of case 4 was characterized by a prevalence of granulomas with a tendency to confluence **(A)**. The dense histiocytic infiltration accompanied the tumor cells even in a microscopic focus where the tumor capsule was exceeded **(B)**, H&E, x20.

All foci had discrete granuloma formations, except one with confluent granulomatous inflammation (Case 4, Figure 2).

**Figure 3:**
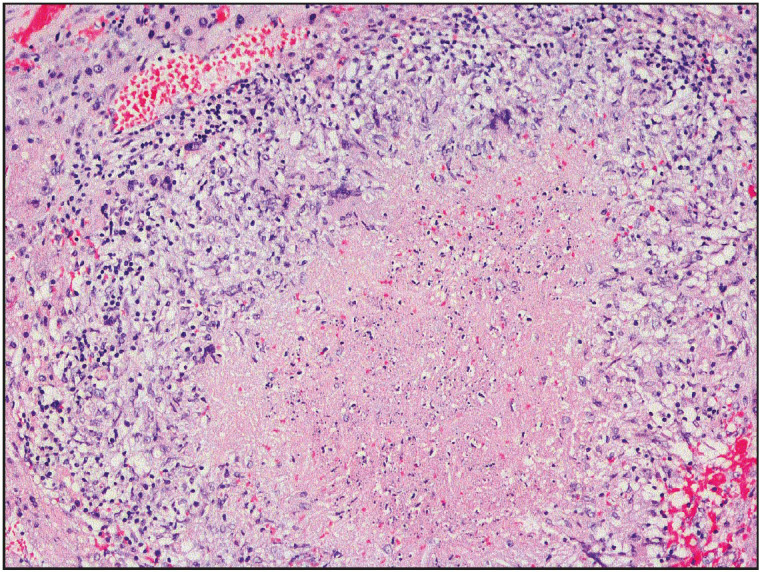
Necrotizing epithelioid cell granulomas with palisading histiocytes and multinucleated giant cells (One focus of Case 5), H&E, x40.

Only 1 case revealed necrotizing granulomas with palisading histiocytes (Case 5, Figure 3). Langhans-type giant cells were common and identified in 6 of 9 foci.

The moderate density of lymphocytic infiltration was intermingled with histiocytes in all granulomas. No eosino-phils were identified. Granulomas did not contain any tumor cells, either.

Two tumor foci revealed rare granulomas in the adjacent liver parenchyma (Case 3 and 4). Lymph nodes were dissected in only one patient (Case 1) which did not reveal any granulomas. No acid-resistant bacilli were identified with Ziehl Neelsen staining.

### Clinical Course

Follow-up and overall survival times (min-max: 1-60 months) were available in all cases. Nine patients died peri-operatively.

Of the remaining 49 patients, 44 were non-granulomatous cases. Since only 4 patients died in this group, and the data of 9% (4/44) does not allow the Kaplan-Meier analysis, median survival could not be calculated. Cases without granulomas had 1-yr, 3-yr and 5-yr survival rates of 89.4%. One of them was alive with multiple intraabdominal recurrences at 29th month.

Among cases with granulomas (n = 5), 2 died of disease. Median survival was 33.6 months. One of them was alive with the humerus metastasis at 18th month.

Cases with granulomas had 1-yr survival of 80% and 3-yr survival of 40%.

**Figure 4:**
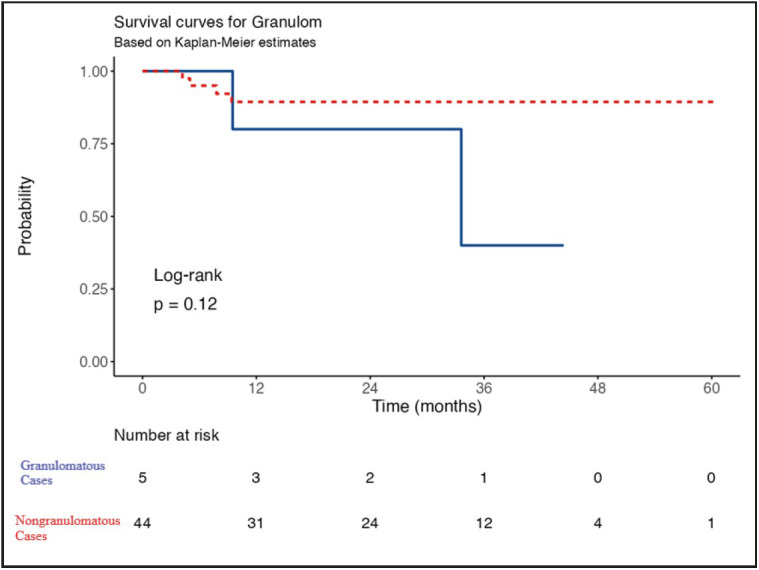
Overall survival comparison of granulomatous vs. nongranulomatous hepatocellular carcinoma cases.

The overall survival was not found to be different between the groups (p = 0.12) (Figure 4).

## DISCUSSION

Among the 58 HCCs included in this study, which to our knowledge represents the first cohort to date analyzed for this purpose, 5 cases (8.6 %) had granulomas. Granulomatous inflammation in HCC appears to be a very rare histologic finding in the literature, reported in middle-aged patients with viral hepatitis and/or cirrhosis (Table 3) (9–12). There are also 2 more cases not included in the table since there is no detailed information about the patient’s characteristics. One of these is a HCC case with intratumoral granulomas written by Neville et al. (18), and the other is a rare case diagnosed as hepatocellular neoplasm of uncertain malignant potential (19).

**Table 3:** Clinicopathologic features of Hepatocellular Carcinoma Cases without accompanying established granulomatous diseases.

**First Author, Publication year**	**Age, Gender**	**Tumor size**	**Examined material type**	**Tumor grade**	**Fibrotic stage and underlying disease**	**Cellular components of the granuloma**	**Necrosis in granulomas**	**Presence of granuloma in nonneoplastic liver**
Tomimatsu et al. 1982 (9)	55, Male	Entire left lobe and accompanying multiple intrahepatic foci	Autopsy	Poorly differentiated	Cirrhosis	Epithelioid cells, Langhans-type giant cells, and varying numbers of lymphocytes	No necrosis	No
Mourra Flejou, 2001 (10)	54, Male	2 foci (1.7 cm and 0.7 cm)	Segmentectomy	Edmondson’s grade II	Cirrhosis, Hepatitis C	Epithelioid cells and multinucleated Langhans cells	No necrosis	No
Ichikawa et al. 2002 (11)	58, Male	Not mentioned	Subsegmentectomy	Edmondson’s grade II	Not mentioned, Hepatitis B	Epithelioid cells and multinucleated Langhans-type giant cells	No necrosis	Yes
Mizota et al. 2021 (12)	69, Female	Multiple nodules, the largest of which is 81 mm	Partial hepatectomy	Not mentioned	Not cirrhosis, Hepatitis C	Necrotic cells and epithelioid cell granulomas with multinucleated giant cells	Present	Not mentioned

Granulomas accompanying malignancy can be located within tumor stroma in a randomly dispersed fashion. They can also show tendency to the peripheral regions of neoplastic lesion, such as beneath the tumor pseudocapsule in capsulated lesions (but still within the tumor) or at the edge of tumor stroma, creating a border between neoplasia and surrounding parenchyma (7,20–22). The latter was the predominant pattern detected in this cohort (6 of 9 foci). Bässler and Birke reported lymphocyte-poor (naked) and compact ‘sarcoid-like’ granulomas relatively in circumferential regions (21). Since lymphocytes were accompanying the histiocytes in all granulomas, no sarcoid-like granulomas were detected in this study and it was not possible to comment on such a distribution difference.

In addition to tumor stroma, granulomas may also be detected in the nonneoplastic parenchymal part of the tumorous organ (22). This phenomenon was seen in only 2 of 58 cases (Case 3 and 4).

The granulomas were not fairly uniform. There were predominantly non-necrotizing (7 foci, 4 cases) and less frequently necrotizing ones (2 foci, 1 case), some with palisading histiocytes (1 foci), similar to the infrequent reporting of necrotizing granulomas in the literature (23). All of them had a mononuclear infiltrate as reported in literature (21). However, none of them had eosinophils, unlike Kojima et al.’s findings (24).

When it comes to the underlying mechanism of granuloma formation, T cell-dependent reaction to degrade tumor particles is the recognized pathogenesis although the exact antigens in each type of tumor are not known (25). In our opinion, their propensity to locate circumferentially as well as the conspicuous alignment around inner nodule of one case (Case 5) are supportive histologic features of the host reply to tumor antigens.

Soluble tumor antigens also induce a granulomatous response wherever they drain (i.e., regional LNs, liver) (3,25). This cohort revealed only one example for granulomatous lymphadenitis (in a case w/o granulomas in his tumor) and 4 cases with rare granulomas in the nonneoplastic liver (2 cases w/o granulomas and 2 cases with granulomas in their tumor - Case 3 and 4).

Specific to necrotizing granulomas, two additional mechanisms are also discussed. Bassler and Birke interpreted necrosis as a hypersensitivity reaction triggered by persistent antigen expression due to their patient’s recurrence history of breast carcinoma (21). And since nonviable tumor cells were detected in necrotizing granulomas, Coyne pointed out the role of necrotic tumor cells (23). However, the one necrotizing case (Case 5) in this series had neither a previous history of malignancy nor necrotic tumor cells in the granulomas.

The cytoplasmic content of tumor cells is another suspected reason of granulomatous reaction. The high glycogen content seen in seminomas, and clear cell and papillary renal cell carcinomas is stated as a trigger of this reaction. The striking accumulation of granulomas in close relation with steatohepatitic tumor cells appears to support this mechanism (Case 1 and Case 4) as well as clear cells in Case 3. In this regard, Kai et al. reported an unusual HCC case with dense fat-loaded histiocyte infiltration, suggesting the role of lipid in tumor cells driving macrophages for phagocytosis. High levels of chemokines and colony-stimulating factors for macrophages were detected in their case as supportive of this pathogenesis (26).

Drugs are also reported as a causative factor of granulomas in hepatocellular lesions. Ichikawa et al. recorded chemo embolic lipiodol droplets, while others reported the role of oral contraceptives (OC) (11,27). None of the granulomatous cases had neoadjuvant therapy or OC history in this study.

Bieze et al. reported 5 hepatocellular adenomas (HCA) with granulomas in the tumor stroma and/or background liver tissue. Since 4 of them were inflammatory type HCA, they pointed to the impact of prolonged chronic inflammatory stress (27). Unlike this report, the inflammatory infiltrate score was minimal for 4 of 5 cases except Case 4 with moderate/dense infiltration, with no statistically significant difference between the groups (p>0.05).

It is worth noting that since granulomatous inflammation has a wide range of etiology, their presence in a malignant case raises the question of exclusion of the other reasons to argue the aforementioned pathogenesis. The differential diagnosis can be extremely difficult, especially in cases with systemic granulomatous response known as ‘tumor-related sarcoid-like reaction’ (7,28). In this cohort, none of the patients had a history of any other granulomatous disease not only before the surgery but also along the follow up period, except one granulomatous case had a history of Tbc, which had been effectively treated 7 years ago (Case 1). In Case 1, the small, noncaseating structure of the granuloma, and additionally and more importantly the presence of accompanying giant cells in its immediate vicinity and concentration of this histiocytic reaction solely to the steatotic area of tumor, were the most suggestive features that it was not associated with Tbc (Figure 1A). The hilar lymph nodes of the explant were also devoid of any granulomas, which minimizes the possibility of systemic/infection related inflammatory response. Besides, the patient had no clinical findings for Tbc.

The prognostic impact of the granulomas remains an unanswered question that has recently generated remarkable interest among cancer researchers, especially with the introduction of immune checkpoint inhibitors (ICIs) (22). Despite some previous conflicting reports, there is progressively increasing literature indicating its role in local tumor regression as well as metastasis prevention (21,29). Recently, sarcoidosis-like granulomatous reactions (SLR)*- similar to sarcoidosis in aspects of both histology and clinical manifestation -* have been described as a side effect of ICIs. There is evidence that these drugs show their effect as an anti-tumor agent through this reaction (30). They inactivate proteins (synthesized by immune cells or tumor cells) that inhibit antitumor T cell activity (31). In several malignancies, comparison of patients with and without SLR supports an association between this reaction and a better clinical response, as well as better overall survival (32). Our findings were open to interpretation in either way in terms of prognostic effect of granulomas. Although no overall survival difference was identified in this study, the granulomatous cases had worse survival rates. On the other hand, they were associated with better prognostic factors. Higher grade (3-4/4), macrovascular invasion, and relatively larger tumor size were identified in the non- granulomatous group.

In summary, hepatocellular carcinomas with granuloma-tous inflammation are not a very rare finding (8.6%), discovered in smaller tumors with lower grades (Grade 1-2). Granulomas are usually the non-necrotizing type. Their peripheral location within tumor stroma and relation to clear cells and steatohepatitic tumor areas are remarkable. Clinicopathologic features and prognosis appear to be similar to hepatocellular carcinomas without granulomas, although definitive interpretation is not possible due to the limited numbers and follow-up time. Since the number of patients was low, these non-significant results may not represent a true indifference.

This study suggests the interference of the host immune system against tumor antigens and the role of cytoplasmic content. However, further studies are needed to elucidate the underlying mechanisms of this reaction type and to establish the clinical impact.

## Conflict of Interest

All authors declare that they have no conflict of interest.
